# Comparison of Oropharyngeal Microbiota from Children with Asthma and Cystic Fibrosis

**DOI:** 10.1155/2017/5047403

**Published:** 2017-12-27

**Authors:** Sébastien Boutin, Martin Depner, Mirjam Stahl, Simon Y. Graeber, Susanne A. Dittrich, Antje Legatzki, Erika von Mutius, Marcus Mall, Alexander H. Dalpke

**Affiliations:** ^1^Department of Infectious Diseases, Medical Microbiology and Hygiene, University Hospital Heidelberg, Heidelberg, Germany; ^2^Translational Lung Research Center Heidelberg (TLRC), German Center for Lung Research (DZL), Heidelberg, Germany; ^3^Dr. von Hauner Children's Hospital, LMU Munich, Munich, Germany; ^4^Division of Pediatric Pulmonology and Allergy and Cystic Fibrosis Center, Department of Pediatrics, University Hospital Heidelberg, Heidelberg, Germany; ^5^Department of Translational Pulmonology, University of Heidelberg, Heidelberg, Germany; ^6^Department of Pneumology and Critical Care Medicine, Thoraxklinik, University Hospital Heidelberg, Heidelberg, Germany

## Abstract

A genuine microbiota resides in the lungs which emanates from the colonization by the oropharyngeal microbiota. Changes in the oropharyngeal microbiota might be the source of dysbiosis observed in the lower airways in patients suffering from asthma or cystic fibrosis (CF). To examine this hypothesis, we compared the throat microbiota from healthy children (*n* = 62) and that from children with asthma (*n* = 27) and CF (*n* = 57) aged 6 to 12 years using 16S rRNA amplicon sequencing. Our results show high levels of similarities between healthy controls and children with asthma and CF revealing the existence of a core microbiome represented by *Prevotella*, *Streptococcus*, *Neisseria*, *Veillonella*, and *Haemophilus*. However, in CF, the global diversity, the bacterial load, and abundances of 53 OTUs were significantly reduced, whereas abundances of 6 OTUs representing opportunistic pathogens such as *Pseudomonas*, *Staphylococcus*, and *Streptococcus* were increased compared to those in healthy controls controls and asthmatics. Our data reveal a core microbiome in the throat of healthy children that persists in asthma and CF indicating shared host regulation favoring growth of commensals. Furthermore, we provide evidence for dysbiosis with a decrease in diversity and biomass associated with the presence of known pathogens consistent with impaired host defense in children with CF.

## 1. Introduction

Since their emergence, prokaryotes colonize all niches from the extremophilic ones to eukaryotic hosts. One of those niches, long time considered sterile, is the lower airways and the lungs of humans [1]. Of note, even a century ago, it was acknowledged that the lungs are under constant exposure to microorganisms contained in inhaled air and the upper respiratory tract [2]. The conclusion of sterility of the lower airways was based on negative results from standard microbiology that however favors the growth of pathogenic bacteria and was not designed to capture the full spectrum of bacterial species (especially anaerobes) [1].

Airway microbiology is still at the beginning of being deciphered, yet with the advances of research and next-generation sequencing, it is now established that the lower airways in healthy subjects are colonized by bacteria from the oropharyngeal microbiota dominated by members of the Firmicutes, Bacteroidetes, and Proteobacteria phyla [1]. Those findings were the keystone of a theory on the acquisition of the airways' microbiome which is based on the island model: the lower airways' (“islands”) microbiota is the result of the colonization from the upper airways (“mainland”) regulated by the elimination from the host and conditions of regional growth [3, 4]. In a healthy subject, the balance between colonization and elimination leads to a neutral equilibrium where the most abundant microbes from the upper airways are the most frequently found bacteria in the lower airways [5].

With the advances of high-throughput sequencing, the view of lung microbiology shifted from a pathogen-centered view to a more global view of the whole microbiome [6, 7]. One of the most promising fields in lung research is to understand the crosstalk between the lung microbiota and respiratory epithelial surfaces especially in inflammatory airway diseases. Changes in the microbiome are found in several lung diseases associated with chronic airway inflammation including COPD, asthma, and CF [8–13]. In CF, by the age of 6 years, most children have experienced a bacterial infection with *H. influenza*, *S. aureus*, or *P. aeruginosa* [14–16]. In younger children with CF, the lower airways' microbiota is quite similar to the one observed in healthy subjects and is a subpopulation of the upper airways' microbiota [8, 17]. In asthma, it is currently under debate how bacterial and viral infections contribute to the increase in airway inflammation. Furthermore, it has been shown that environmental microbial exposures prevent the onset of disease [9, 10, 18]. Yet there is also evidence that microbiota in asthma patients shows signs of dysbiosis compared to that in healthy subjects with an increase in Proteobacteria, particularly *Moraxella* sp., and a decrease in Bacteroidetes, particularly *Prevotella* spp. [9, 10].

In children, it is difficult to get samples from the lower airways because they do not expectorate sputum and because sampling the lower airways by bronchial alveolar lavage is relatively invasive which makes it ethically not permissible except in extreme cases. Therefore, studies in children use oropharyngeal samples as a proxy sampling for lung microbiota. It was shown in healthy subjects and young children with CF that this sampling procedure reflects the lower airways' microbiome; based on the island model, the throat microbiota is even the source of microbial colonization [3, 5, 8, 17]. Therefore, the hypothesis of our study was to decipher if children with asthma and CF differ from healthy children regarding throat microbiota and if they differ from each other. To test that hypothesis, we compared the throat microbiota of healthy school-age children (6–12 y) with that of age-matched children with asthma and CF.

## 2. Materials and Methods

### 2.1. Subjects

This study was performed with throat swab samples from healthy school-age children and age-matched children with asthma and CF (Table 1). Children with CF were examined and sampled in the CF center in Heidelberg as approved by the Ethics Committee of the University of Heidelberg, and informed written consent was obtained from the patients, their parents, or legal guardians. The diagnosis of CF was based on the established diagnostic criteria [19]. Healthy controls and children with asthma were part of the cross-sectional GABRIELA study. The GABRIELA study was approved by the ethics committees of the participating universities and the regional data protection authorities. Asthma was defined as either (i) parent-reported wheeze during the last 12 months at two different time points of the study, (ii) when a positive answer a positive answer to the question “Did your child ever use an asthma spray?” was given, or (iii) when a doctor diagnosed asthma at least once or wheezy bronchitis more than once. Healthy controls were defined by the absence of all those indications (i.e., no doctor's diagnosis, no use of inhaler spray, and no wheeze at the two time points of the study).

### 2.2. Sample Collection and Storage

Airway samples from CF patients were obtained during routine visits at the CF center with oropharyngeal Eswabs (BD ESwab Collection Kit, Becton Dickinson, Heidelberg, Germany). Samples were treated in the first 24 h with PMA™ dye (Biotium Inc., Hayward, USA) to remove DNA from dead bacteria as previously described [8] and stored until DNA extraction at −20°C. A supplementary analysis was performed to evaluate the effect due to PMA treatment on a subset of the CF cohort (with and without PMA), and no significant differences in biomass and beta and alpha diversity were observed (Suppl. Figures 1 and 2). Throat swabs from asthmatic and control children were collected using sterile dry cotton-headed swabs (MASTASWAB MD 559, MAST Diagnostica GmbH, Germany). After sampling, the swab was immediately placed back in the collection tube and stored within 24 h at −20°C. All DNA extractions were performed using the QIAamp Mini Kit (Qiagen, Hilden, Germany). Protease solution (7.2 mAU) and 200 *μ*L of Buffer AL were added to the sample followed by a 15 sec vortex. Samples were incubated at 56°C for 10 min and then purified according to the manufacturer's protocol. DNA was eluted by adding 100 *μ*L of buffer AE to the column, incubated for 1 min at room temperature, and centrifuged at 6000 ×g for 1 min. Negative controls were performed by doing the extraction without clinical samples.

### 2.3. Microbiome Analysis

DNA was amplified using universal bacterial primers flanking the V4 region (515F and 806R [20]). PCR was performed with numerous controls (positive control with a mock community (HM-782D, BEI Resources, Manassas, USA) and negative control of the PCR and extraction methods) to exclude contaminations [8]. Negative controls for both PCR and extraction did not yield any quantifiable amplicons. PCR products were ligated to the sequencing adapters and paired-end sequenced on an Illumina MiSeq system (250 cycles). Raw sequences were processed to remove low-quality reads and chimera. Sequences were subsampled to obtain the same number of reads per sample and then clustered as operational taxonomic units (OTU) (using the threshold of 3% of divergence). OTUs were classified at the taxonomic levels by comparison with sequences from the SILVA database using Mothur [21]. 5,312,076 of nonchimeric good-quality reads were obtained and subsampled to 7163 reads per sample to normalize the effort of sampling for every sample (Good's coverage of 99.1% (95.2–99.8)). The sequencing of a mock community with known species allowed us to calculate the overall error rate of the PCR and sequencing methodology. This error rate was 1.56 × 10^−5^. Furthermore, we found 19 OTUs with abundance higher than 1% in the mock community when we were expecting 20. The taxonomical assignment of those OTUs showed a matching identity to the 20 expected species. Only two *Staphylococcus* species were clustered together in the same OTU due to identical 16S rRNA V4 sequences between *S. aureus* and *S. epidermidis*. The mean distance based on the Morisita-Horn index between the different mock communities used in different sequencing runs was 0.032.

Quantitative PCR (qPCR) was used to evaluate the number of 16S copies as a proxy measure of biomass. qPCR was performed using the Unibac primer (forward: 5′-TGG AGC ATG TGG TTT AAT TCG A-3′; reverse: 5′-TGC GGG ACT TAA CCC AAC A-3′) as previously described [8].

### 2.4. Statistical Analyses

The OTU table with all OTUs (normalized by subsampling 7163 reads per sample) obtained was used to calculate descriptive indices for alpha diversity (nonparametric Shannon index), richness (Chao1 richness estimate), and evenness (Shannon index-based measure of evenness). Variation in the alpha diversity and clinical parameters was tested with a pairwise Wilcoxon sum rank test. Beta diversity variations were evaluated at the OTU level via Principal Coordinate Analysis (PCoA) and PERMANOVA based on the Morisita-Horn similarity index [22]. *R* ^2^ indicating the strength of the explanatory variable on the distance between the microbiota is reported. Associations with lung function were analyzed by linear regression for continuous variables (*R* ^2^ indicating the strength of the correlation) or the pairwise Wilcoxon sum rank test for categorical variables.

We also performed an analysis with a model based on a negative binomial distribution (DESeq) [23] to detect differentially abundant OTUs between the groups (asthma versus CF, asthma versus healthy, and CF versus healthy). Adjustment for multiple testing was done using the Bonferroni-Hochberg method. All statistical analyses were performed with Mothur 1.37.4 and R 3.3.0 (mainly packages Phyloseq [24] to handle microbiome data, Vegan [25], and DESeq2 [23] for analysis). Scripts for Mothur, R, and the sequence data are deposited in figshare (https://figshare.com/s/1e69261612f3dfcacf42).

## 3. Results

### 3.1. A Decrease in Lung Function and Higher Antibiotic Usage Are Characteristic of the CF Cohort

Children with CF showed a decreased lung function with a FEV1 *z*-score of −1.78 ± 1.37 (versus −0.43 ± 0.79 for asthma (*p* value < 0.001) and −0.44 ± 1.08 for control (*p* value < 0.001)), a FVC *z*-score of −1.42 ± 1.32 (versus 0.12 ± 0.93 for asthma (*p* value < 0.001) and −0.17 ± 0.79 for control (*p* value < 0.001)), and a higher frequency of antibiotic usage (CF versus control: odds ratio = 47.81 (*p* value < 0.001) and CF versus asthma: odds ratio = 21.81 (*p* value < 0.001)) (Table 1).

### 3.2. Comparison of Oropharyngeal Microbiota Reveals Only Minor Shifts between Healthy Children and Children with Asthma or CF

**Figure 1:**
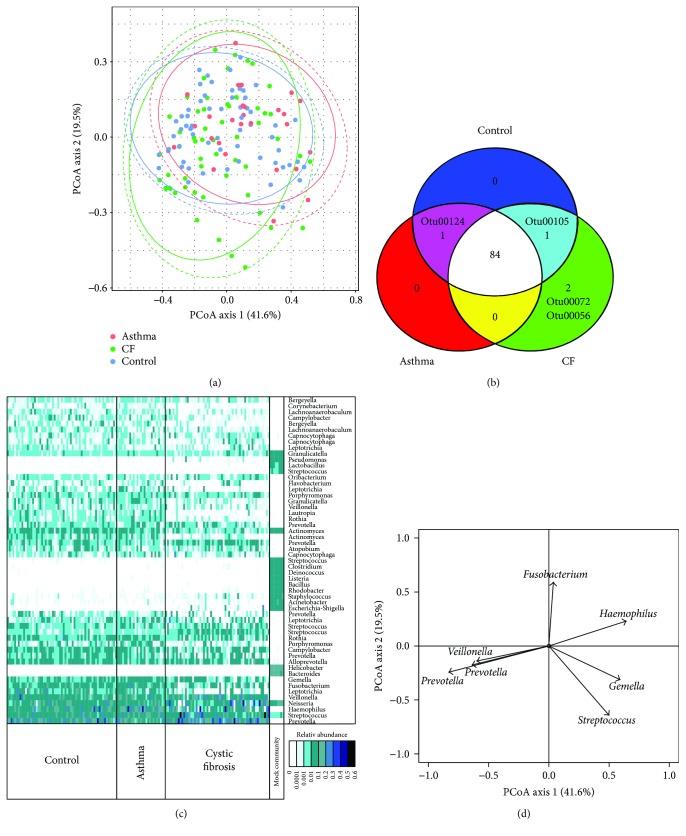
Structure of the throat microbiome of healthy children, children with asthma, and children with CF. (a) Microbial compositions of the whole microbiota were visualized by PCoA. The lines represent the 95% confidence interval assuming a multivariate *t*-distribution (full lines) or a multivariate normal distribution (dashed lines). (b) The presence/absence of the 88 most abundant bacteria (>0.1% relative abundance) was compared between the three groups and is displayed as a Venn diagram. (c) Relative abundance of the 55 most abundant OTUs in each sample clustered according to the cohorts. The mock community was an assembly of DNA from 20 known species with equimolar ribosomal RNA operon counts (100,000 copies per organism per *μ*L). (d) Correlation plots of the major OTUs with the two first axes of the PCoA.

The structure of the whole microbial community was compared using the Morisita-Horn similarity index and PERMANOVA analysis. Although significant differences between the three groups (CF versus control: *R* ^2^ = 0.05 (*p* value < 0.01), CF versus asthma: *R* ^2^ = 0.09 (*p* value < 0.01), and control versus asthma: *R* ^2^ = 0.05 (*p* value < 0.01)) were found, the low values of the *R* ^2^ (indicating the strength of the impact of the disease status on the differences between the microbial structure) and the major overlap of the three cohorts on the PCoA (Figure 1(a)) argue for only minor changes within the structure of the microbiome. This similarity is confirmed by the shared presence of the most common OTUs in each cohort (84 OTUs shared) (Figure 1(b)). Only 2 abundant OTUs were only present in CF patients; those belonged to the genus *Pseudomonas* and *Phyllobacterium*. One OTU belonging to the genus *Moraxella* was absent from the CF group, and one belonging to the genus *Aggregatibacter* was absent from the asthma groups. The difference between the three cohorts was mostly due to the modulation of the abundance of the most dominant genera and variation in the less abundant genera rather than to extinction of specific genera (Figure 1(c)). The CF cohort compared to controls and asthmatics showed an increasing trend in four dominant genera: *Prevotella*, *Neisseria*, *Veillonella*, and *Streptococcus*, while asthmatic children tended to have higher *Haemophilus* abundance compared to CF and controls.

The distribution over the two axes of the PCoA relied on differences in the following major components of the microbiota in the throat: OTUs belonging to the following genera *Prevotella*, *Veillonella*, *Streptococcus*, *Gemella*, *Haemophilus*, and *Fusobacterium* (Figure 1(d)). Samples from asthmatic patients were showing a significantly higher value on axis 1 of the PCoA compared to those from CF patients (*p* value < 0.01) and healthy people (*p* value < 0.05) indicating a higher abundance of the OTUs (mainly *Gemella* and *Haemophilus*) correlating positively with this axis of the PCoA and a lower abundance of OTUs belonging to *Prevotella* and *Veillonella* correlating negatively with axis 1. Gender and antibiotic usage within 4 weeks prior to sampling were also significantly affecting the microbiota structure of the full population (independent of the cohort) but with a small *R* ^2^ (gender: *R* ^2^ = 0.03 (*p* value < 0.01) and antibiotic usage: *R* ^2^ = 0.01 (*p* value < 0.01)).

When the cohort was analyzed separately, only the gender was affecting the CF cohort (*R* ^2^ = 0.07 (*p* value = 0.02)) and no antibiotic usage effect was observed. For the control and asthma cohort, no effects were observed for gender and antibiotic usage but the low prevalence of antibiotic usage did not allow strong statistical evaluation.

### 3.3. Oropharyngeal Microbiota from Children with CF Shows a Decrease in Alpha Diversity and Biomass

**Figure 2:**
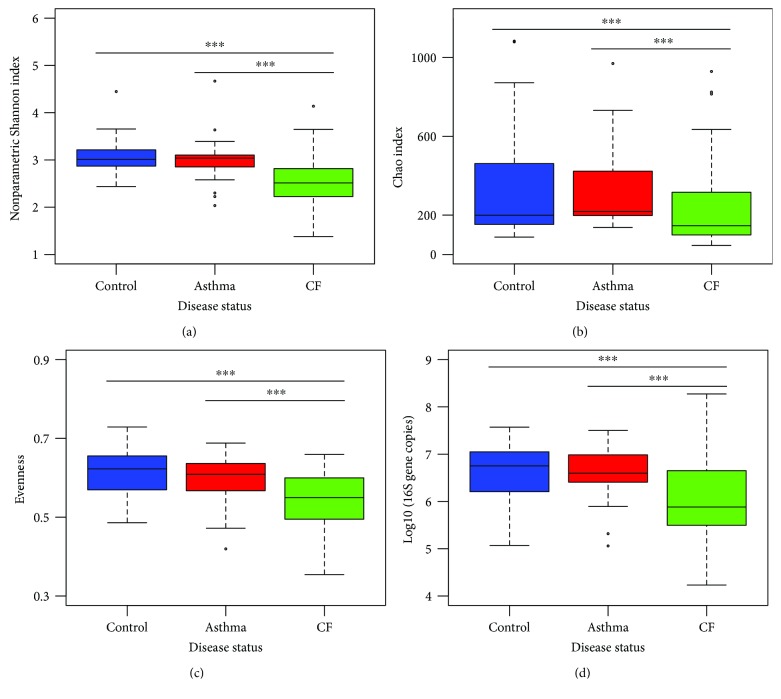
Diversity and biomass are decreased in the oropharyngeal microbiome of children with CF. (a) *α*-Diversity was assessed by the nonparametric Shannon index. (b) Richness was estimated by the Chao index. (c) Evenness was derived from a Shannon index-based measure of evenness. (d) Global biomass measured as the number of 16S rRNA gene copies. Statistical significance was calculated by the Wilcoxon test. ^∗∗∗^ *p* value < 0.001.

Microbiota of the throat from children with asthma versus control children did not differ in alpha diversity (*p* value = 0.56). However, throat microbiota from children with CF showed strong evidence of a decrease in the global alpha diversity compared to that from asthmatic and control children (*p* value < 0.001) (Figure 2(a)). The change in diversity was due to a difference in the total amount of species present (Figure 2(b)), as well as due to the distribution of the abundance of the species (Figure 2(c)). Microbiota from children with CF was less diverse and less even indicating the overgrowth by one or few species. This observation correlated with the increase in the abundance of *Prevotella* and *Streptococcus* in the CF group.

No differences in the numbers of bacteria were observed between asthmatic and control children. However, children with CF harbored less bacteria in their throat (Figure 2(d)). The reduced *α*-diversity is also seen for children with lower lung function, even if only a trend is seen (FEV1 *z*-score: *R* ^2^ = 0.17 (*p* value = 0.06) and FVC *z*-score: *R* ^2^ = 0.17 (*p* value = 0.08)) probably reflecting the impact of CF.

Antibiotic usage within 4 weeks prior to sampling did not influence the alpha diversity within the CF cohort (*p* value = 1). Furthermore, also the nontreated CF samples showed a significantly lower alpha diversity than samples from patients with asthma (*p* value < 0.001) or control samples (*p* value < 0.001) indicating the effect of the disease status even without antibiotic therapy. However, biomass was slightly influenced by antibiotic usage as nontreated CF samples did not show a significant decrease in bacterial load compared to control and asthmatic samples, but treated CF samples showed a significant decrease in biomass. No significant difference in the biomass was observed between treated and nontreated CF samples (Suppl. Figure 3).

### 3.4. Differences in Oropharyngeal Microbiome Composition between Children with CF, Children with Asthma, and Healthy Controls

**Figure 3:**
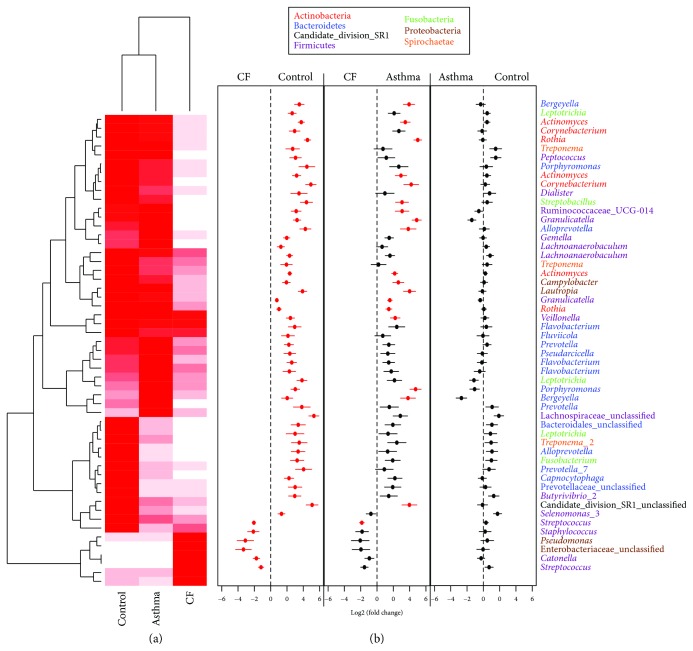
Differences in OTU abundance between healthy children, children with asthma, and children with CF. Differential abundance of each OTU was tested via a method based on the negative binomial distribution and is represented in a heatmap for the significantly differentially abundant OTU. Mean abundance was normalized within each OTU to the maximal values (normalized value_OTU1_ = (relative abundance_OTU1_)/maximum (relative abundance_OTU1_)). The color code is from white (minimal abundance: 0) to red (maximal abundance: 1). Fold changes between the groups and standard errors are displayed in (b). Red dots indicate significant differences after correction for multiple testing. OTUs are named following their genus classification and colored following their phylum classification.

Between children with CF and healthy controls, 53 OTUs were differentially abundant (Figure 3). Most of them were decreased in the CF group except a small group composed of 6 OTUs belonging to *Streptococcus*, *Catonella*, *Enterobacteriaceae*, *Staphylococcus*, and *Pseudomonas*. Interestingly, *Staphylococcus* and *Pseudomonas* are well-known CF pathogens, and the OTUs belonging to *Pseudomonas* were the same as the ones in the mock community indicating that the species is *P. aeruginosa*. The same pattern was observed for the comparison between children with CF and children with asthma with a general decrease in the 20 differentially abundant OTUs in CF except for two OTUs belonging to the *Streptococcus* genus. Despite the visible change in the abundance in the heatmap, no significant differences were observed for the 5 other OTUs that appear more abundant in CF than in controls. This lack of statistical significance might be due to the low number of samples and the higher interindividual variability of those OTUs in the CF cohort.

## 4. Discussion

The aim of this study was to analyze whether differences occur in the throat microbiota relating to inflammatory airway diseases like asthma and CF in comparison to that in healthy children. Our results indicate that the microbiota of CF, asthmatic, and healthy children shows high levels of similarities with a strong core microbiota composed mostly of *Prevotella*, *Streptococcus*, *Neisseria*, *Veillonella*, and *Haemophilus*. The prevalence of those bacteria in the throat microbiota was demonstrated previously in healthy, CF, and asthmatic children indicating a close relationship, but our study is the first to compare the three groups with the same DNA extraction method, primer usage, and sequencing method thus controlling for potential technical bias [8, 9, 17]. Free of those biases, we were able to demonstrate that the CF group showed a decrease in both diversity and total bacterial load in the throat in comparison to asthmatic and control children. The decrease in diversity was due to an increase in the abundance of dominant genera, especially *Prevotella* and *Streptococcus*. The increase in the dominance influences the evenness of the bacterial community and the overall richness as it was observed before in CF and COPD [8, 13]. The decrease in the biomass would also indicate a less abundant microbiome in the throat of CF patients. The CF group also showed a significant increase in typical pathogens in the throat like *Pseudomonas*, *Staphylococcus*, and the atypical pathogen *Phyllobacterium* [26–28]. It could be hypothesized that the lower biomass in CF children compared to asthmatic and healthy children facilitates the colonization in the throat by additional pathogens as it decreases the competition pressure and colonization resistance by protective commensals represented by *Prevotella*, *Neisseria*, *Streptococcus*, and *Veillonella*. This theory is supported by the fact that in CF patients, the latter specific genera are decreasing in the lower airways after colonization by typical pathogens like *Pseudomonas aeruginosa* [8, 29]. The commensal and protective role of core throat bacteria found here and also in healthy subjects is still not elucidated; especially, the specific role of anaerobes in the lower airways remains controversial [30]. However, it was demonstrated that *Prevotella* and other anaerobes exhibit antimicrobial activities and a specific strain of *Prevotella* possessing an altered LPS had less immune-stimulatory activities [31, 32]. Our data cannot exclude the hypothesis that CFTR mutation is modifying the niches and the regional growth for specific CF pathogens in the lungs and that those modifications are the driving force of the establishment of pathogens in the lungs.

The strong similarities between the throat microbiota from healthy children and that from asthmatic children were already demonstrated within a larger cohort in which no differences were detectable between asthma and control children [9]. Our data with a subsample of this study and using a different analysis (DESeq) confirm these findings, indicating the absence of effects of asthma in the throat. Yet, those results are in contradiction with those of other studies with smaller cohorts [33, 34], in which a significant increase in *Haemophilus* and a decrease in *Prevotella* were seen in asthmatic children. On closer examination, our data show a similar trend, yet overall no severe or significant dysbiosis in the examined age group was observed. In our setting, statistical tests did not indicate significance. The analysis with a model based on a negative binomial distribution (DESeq) is less sensitive to false positives than Metastat or a generalized linear model analysis when applied to a small-size cohort. As shown by Depner et al., we did not observe a difference in the total bacterial load between asthma and control children [9].

The strong overlap and shared core microbiome between the three groups are an evidence of the importance of the regulation of the upper airways' microbiota by the host. As patients with asthma and CF come from different environments and disease status and showed the highly similar microbiota, this indicates a positive selection to conserve the structure of the microbiota. It seems that there is a tight immune regulation of the microbiota in the throat that allows only the growth of commensals or at least nonpathogenic bacteria [35]. However, this regulation seems to be slightly unbalanced in CF patients leading to a decrease in diversity and total amount of bacteria correlating with the increased chance to harbor a typical CF pathogen (*Staphylococcus* or *Pseudomonas*). As we did not observe changes in asthma, the imbalance observed in CF could be due to impaired host defense mechanisms associated with CFTR dysfunction in the airways, changes in regional growth condition in the lungs, and/or CF-specific antibiotic regimen [36–38].

Our study focused on oropharyngeal swabs. Yet in the light of recent results on healthy, asthmatic, and CF children and the current theory on acquisition and establishment of the airway microbiome, the throat microbiota will have a direct influence on the lower airways and therefore on the colonization probability [4, 8, 10, 17]. Therefore, our findings might indicate that the core microbiome found between the three cohorts can be the source of commensals that will colonize the lower airways. The colonization by those commensals is regulated by the host in a complex balance between migration and elimination [4]. In this context, a lower migration of protective commensals may increase the chance of pathogenic colonization in the lungs for CF [39]. This correlates with the finding that a decrease in diversity in the lower airways of CF patients was linked to a higher chance of further infection and a more severe inflammatory response [16, 40]. However, as our study did not analyze lower airways' samples, we can only speculate that the core microbiome found in the three cohorts and the slight differences specific to CF also occur in the lower airways.

The limitation of our study is that despite the common DNA extraction method, primer choice, and sequencing technology used, there are still some differences in the methodology applied to the CF cohort and the two other cohorts. One methodological discrepancy was the use of different swabs and PMA treatment that might affect both diversity and biomass. However, we showed by comparing 10 samples with and without PMA that, in our study cohort, PMA did not significantly affect both biomass and diversity (Suppl. Figures 1 and 2). Finally, the two cohort sampling methods used different types of swabs that might influence the sampling of microbial community; therefore, this bias still has to be taken in consideration. However, the most important technical bias in microbiota studies are the DNA extraction method, primer choice, and sequencing technology, and therefore, in this study using the same primers and DNA extraction and sequencing technology, we abrogated most of the major bias [41–43]. Furthermore, the sampling size of the asthmatic cohort was smaller compared to that of the CF and control cohort; thus, differences between this cohort and the two others have to be interpreted carefully.

In conclusion, this study analyzed for the first time the throat microbiota of healthy school-age children and children with asthma and CF with comparable methodology. Our results show that the three patient groups showed a high level of similarity indicating a core microbiome and host regulation that favors the growth of commensals. However, the CF group showed a decreased diversity and biomass of the microbiota associated with the presence of known CF pathogens consistent with impaired host defenses associated with CFTR malfunction in the airways.

## Figures and Tables

**Table 1:** Demographic description of the cohort.

	Control	Asthma	CF
*n*	62	27	57
Male/female	28/34	21/6	46/11
Age in years (min–max)	10.10 (8–12)	10.00 (8–12)	10.61 (6–12)
FEV1 in L (min–max)	1.99 (1.15–3.18)	1.83 (1.26–2.35)	1.92 (0.88–3.34)
FEV1 *z*-score ± SD	−0.44 ± 1.08	−0.43 ± 0.79	−1.78 ± 1.37
FVC in L (min–max)	2.36 (1.44–3.87)	2.25 (1.53–3.34)	2.40 (1.04–3.86)
FVC *z*-score ± SD	−0.17 ± 0.79	0.12 ± 0.93	−1.42 ± 1.32
% antibiotic use within 4 weeks prior to sampling	1.61	3.70	45.61

FEV1: forced expiratory volume in 1 second; FVC: forced vital capacity. *z*-scores were calculated following the Global Lung Function Initiative (GLI) equation from 2012 [44].

## Supplementary Materials

Supplementary Materials Supplementary Figure 1: PMA treatment of the DNA did influence neither diversity nor biomass of the CF microbiome in the analyzed cohort. A subset of samples (*n* = 10) from the CF cohort was analyzed for changes in diversity and global biomass with and without PMA treatment. No significant differences were observed. Supplementary Figure 2: PMA treatment of the DNA did not influence the structure of the CF microbiome in the analyzed cohort. A subset of samples (*n* = 10) from the CF cohort was analyzed for changes in the microbial structure with and without PMA treatment. No significant differences in the structure of the microbiome were observed. Paired samples are in the same color. The lines represent the 95% confidence interval assuming a multivariate *t*-distribution (full lines) or a multivariate normal distribution (dashed lines). Supplementary Figure 3: impact of antibiotic therapy prior to sampling on the diversity and biomass of the microbiome in the analyzed cohort. Samples were classified in two groups “treated” and “nontreated” based on the usage of antibiotic within 4 weeks prior to sampling. Statistical significance was calculated by the pairwise Wilcoxon test. ^∗∗∗^ *p* value < 0.001.
