# Supplementary material for: Comparison of Oropharyngeal Microbiota from Children with Asthma and Cystic Fibrosis
Source: Mediators Inflamm. 2017 Dec 27;2017:5047403. doi: 10.1155/2017/5047403 (PMC5763206; doi:10.1155/2017/5047403)

Supplementary Figure 1: *PMA treatment of the DNA did neither influence diversity nor biomass of the CF microbiome in the analyzed cohort.*

A subset of samples (n=10) from the CF cohort was analyzed for changes in diversity and global biomass with and without PMA treatment. No significant differences were observed.

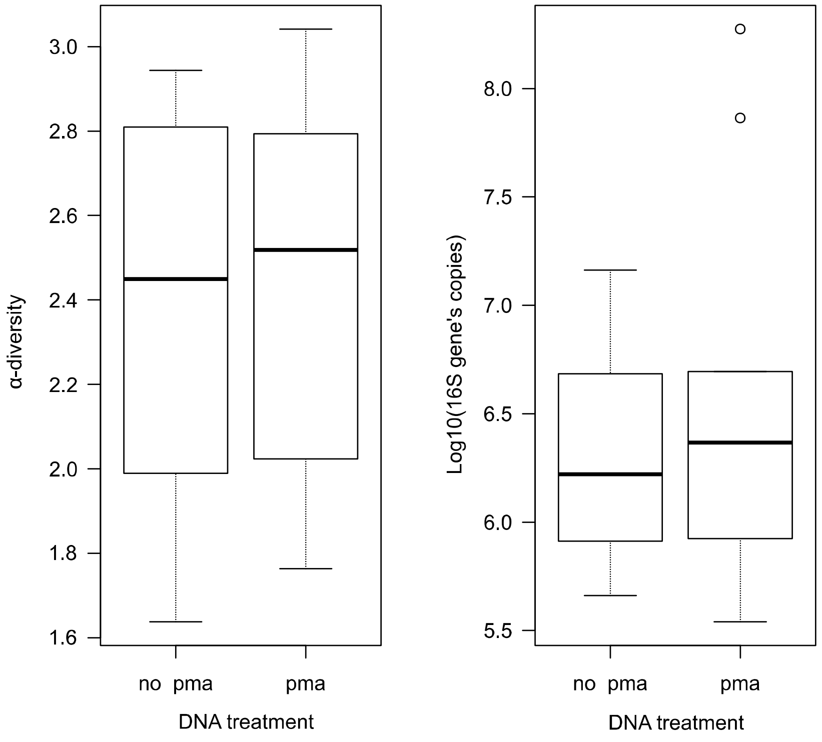

Supplementary Figure 2: *PMA treatment of the DNA did not influence the structure of the CF microbiome in the analyzed cohort.*

A subset of samples (n=10) from the CF cohort was analyzed for changes in the microbial structure with and without PMA treatment. No significant differences in the structure of the microbiome were observed. Paired samples are in the same color. The ellipses represent the 95% confidence interval assuming assuming a multivariate t-distribution (full lines) or a multivariate normal distribution (dashed lines).

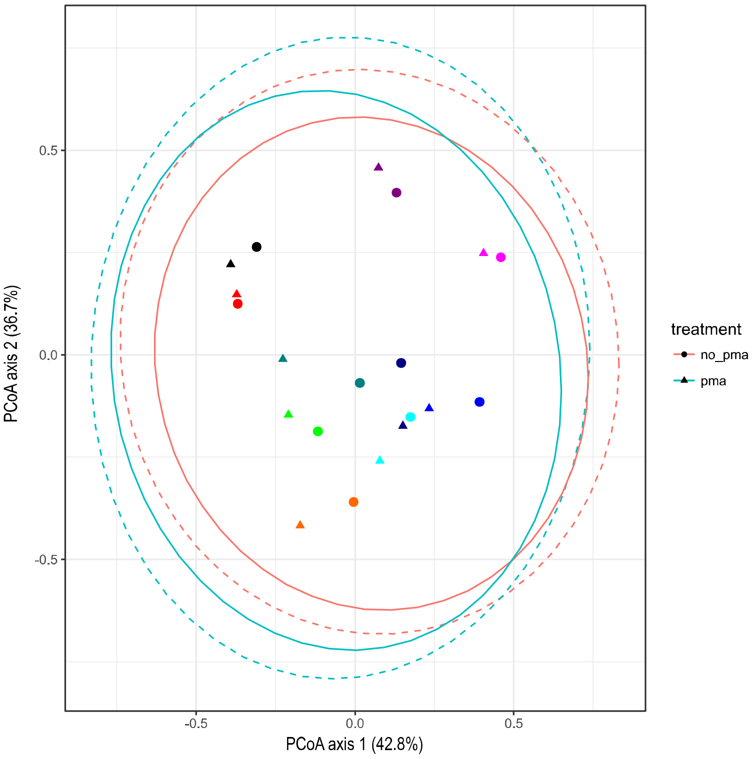

Supplementary Figure 3: *Impact of antibiotic therapy prior of sampling on the diversity and biomass of the microbiome in the analyzed cohort.*

Samples were classified in two groups ‘treated’ and ‘non treated’ based on the usage of antibiotic within 4 weeks prior to sampling. Statistical significance was calculated by pairwise Wilcoxon test. P-value code: ***<0.001.

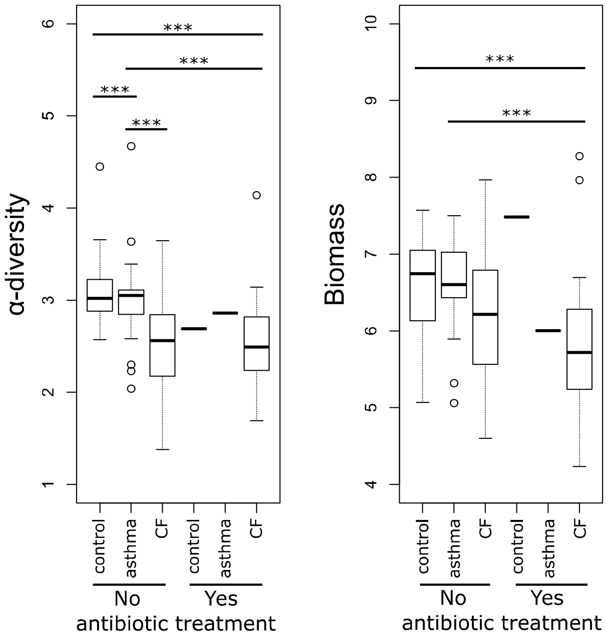

Supplement: Supplementary Materials — Supplementary Figure 1: PMA treatment of the DNA did influence neither diversity nor biomass of the CF microbiome in the analyzed cohort. A subset of samples (n = 10) from the CF cohort was analyzed for changes in diversity and global biomass with and without PMA treatment. No significant differences were observed. Supplementary Figure 2: PMA treatment of the DNA did not influence the structure of the CF microbiome in the analyzed cohort. A subset of samples (n = 10) from the CF cohort was analyzed for changes in the microbial structure with and without PMA treatment. No significant differences in the structure of the microbiome were observed. Paired samples are in the same color. The lines represent the 95% confidence interval assuming a multivariate t-distribution (full lines) or a multivariate normal distribution (dashed lines). Supplementary Figure 3: impact of antibiotic therapy prior to sampling on the diversity and biomass of the microbiome in the analyzed cohort. Samples were classified in two groups “treated” and “nontreated” based on the usage of antibiotic within 4 weeks prior to sampling. Statistical significance was calculated by the pairwise Wilcoxon test. ∗∗∗ p value < 0.001. [file 5047403.f1.pdf]
